# The influence of spirituality, religiosity, and self-care on well-being among Thai infection preventionists during the COVID-19 pandemic

**DOI:** 10.1017/ash.2023.510

**Published:** 2024-02-19

**Authors:** Anucha Apisarnthanarak, M. Todd Greene, Kristin M. Collier, Nongyao Kasatpibal, Karen E. Fowler, Sanjay Saint

**Affiliations:** 1 Division of Infectious Diseases, Thammasart University Hospital, Khlong Luang, Pratum Thani, Thailand; 2 VA Ann Arbor Center for Clinical Management Research, VA Ann Arbor Healthcare System, Ann Arbor, MI, USA; 3 Department of Internal Medicine, University of Michigan Medical School, Ann Arbor, MI, USA; 4 Division of Nursing Science, Faculty of Nursing, Chiang Mai University, Chiang Mai, Thailand; 5 Epidemiology Research Center of Infectious Disease (ERCID), Chiang Mai University, Chiang Mai, Thailand

## Abstract

In a national survey of lead infection preventionists in Thai hospitals, spiritual and religious importance were associated with increased odds of career satisfaction. Cultivating environments for spiritual, religious, and self-care practices within the clinical setting may help facilitate emotional well-being—and prevent burnout—among Thai healthcare workers.

The role of infection preventionist (IP) has gained increased popularity and serves a key role in implementing evidence-based infection prevention practices in Thailand.^1^ The emergence of the COVID-19 pandemic has significantly increased the workload of IPs and recent reports suggest an increasing incidence of burnout among Thai IPs and healthcare workers.^2,3^

Mindfulness, religiosity, and practices such as yoga and meditation may help healthcare workers experience “sacred moments” (defined as “brief periods of time in which people experience a deep interconnectedness that may possess spiritual qualities and emotions”) with patients.^4,5^ These moments may in turn create a sense of joy, peace, and empathy and in doing so, may alleviate stress and burnout during work.^4^ Limited data are available concerning the link between spirituality and measures of well-being among IPs. In this national survey, we assessed the current state of well-being, spirituality, religiosity, and self-care among lead IPs in a sample of Thai hospitals.

## Methods

The Translating Healthcare-Associated Infection Prevention Research into Practice (TRIP) survey instrument, first developed by Krein and colleagues,^6^ has undergone multiple recent revisions and has been deployed in the United States^7^ and several international settings.^2,8^ The surveys assess general hospital, personnel, and infection control program characteristics and examine the regular use of general and specific practices to prevent common hospital infections.

The methods and results for the 2021 TRIP survey distributed in Thailand have been described previously.^2^ Briefly, from 1 February 2021 to 31 August 2021, we surveyed 100 hospitals in Thailand with at least 200 beds and 10 intensive care unit beds. We selected a convenience sample of 20 hospitals within each of the five broad geographic regions (North, South, East, West, and Northeastern) to achieve national representation. The surveys were addressed to the lead IP at each hospital. In addition to the general hospital characteristics and infection prevention-specific questions, the 2021 TRIP survey also assessed how IPs rated importance of spirituality, religiosity, and self-care, as well as measures of well-being.

We generated and examined descriptive statistics (proportions for categorical data and mean ± standard deviation [SD] for continuous variables) for all hospital and infection preventionist-specific characteristics. All measures of spirituality, religiosity, self-care, and well-being were assessed on a 5-point Likert scale. For our analyses, these measures were dichotomized with responses of 4 (agree) or 5 (strongly agree) coded as 1, and 0 otherwise. Multivariable logistic regression models were used to examine the associations between measures of spirituality/religiosity, and self-care and well-being. Dichotomous well-being measures of burnout, uncaring, and career satisfaction were used as primary outcomes. All models were also adjusted for respondent tenure, bed size, medical school affiliation, presence of a hospital epidemiologist, and leadership support for the infection control program.

All analyses were conducted in Stata MP 17.0 (StataCorp, College Station, TX). This study was approved by the Institutional Review Board of the Faculty of Medicine, Thammasat University.

## Results

The lead IP at a total of 100 hospitals were surveyed and all responded (response rate of 100%). Responding IPs were from general, regional, military, university, and private hospitals from all regions of Thailand. Hospital characteristics and spirituality, religiosity, self-care, and well-being characteristics of responding IPs are shown in Table 1. Most IPs felt that spiritual well-being was important for emotional well-being (77%), religious and spiritual beliefs act as a source of comfort and strength during life’s ups and downs (67%), and an organized religious or spiritual community was important (65%). A total of 79% of Thai IPs reported that individual self-care practices such as meditation, yoga, listening to music, exercising, and communing with nature were important. Approximately one-quarter of IPs reported feeling burned out from work (29%) and becoming more uncaring towards people since taking their job as an IP (25%). If given the opportunity to revisit their career choice, a total of 61% of respondents indicated that they would choose to become an IP again.

**Table 1.** Select characteristics

Hospital characteristics	
Hospital bed size (mean (SD))	592.7 (384.6)
Affiliated with a medical school	58%
Presence of a hospital epidemiologist	58%
Good/excellent support from leadership for the infection control program	31%
**Infection preventionist characteristics**	
Number of years in current job position (mean (SD))	8.8 (7.1)
Spiritual well-being is important for one’s emotional well-being	77%
Religious or spiritual beliefs act as a source of comfort and strength during life’s ups and downs	67%
An organized religious or spiritual community is important to me	65%
Individual self-care practices (e.g., meditation, yoga, listening to music, exercising, and nature) are important to me	79%
I feel burned out from my work	29%
I have become more uncaring towards people since I took this job	25%
If given the opportunity to revisit my career choice, I would choose to become an infection preventionist again	61%

Adjusted odds ratios (OR) and 95% confidence intervals (CI) for the spirituality, religiosity, and self-care predictors are shown in Table 2. Statistically significant associations were not detected between any of the primary predictors (spirituality, religiosity, or self-care importance) and the emotional exhaustion (i.e., burnout) or depersonalization (i.e., uncaring) domains of well-being. Conversely, we detected consistent, statistically significant associations between the measures of spirituality and religiosity and the personal accomplishment (i.e., career satisfaction) domain of well-being. Specifically, IPs had increased odds of reporting they would choose to become an IP again if they also reported that spiritual well-being was important (OR = 5.12, 95% CI = 1.80–14.55, *P* = .002), religious or spiritual beliefs act as a source of support and comfort (OR = 3.43, 95% CI = 1.36–8.64, *P* = .009), or an organized religious or spiritual community was important (OR = 4.37, 95% CI = 1.73–11.02, *P* = .002). Although the association between career satisfaction and importance of self-care practices trended in the same direction, the association did not reach statistical significance (OR = 2.56, 95% CI = 0.91–7.24, *P* = .08).

**Table 2.** Multivariable adjusted associations between spirituality, religiosity, and self-care importance and measures of well-being by domain among Thai infection preventionists

Predictor	OR	95% CI	*P*-value
**Emotional exhaustion domain “I feel burned out from my work”**			
Spiritual well-being is important for one’s emotional well-being	0.89	0.31–2.57	.82
Religious or spiritual beliefs act as a source of comfort and strength during life’s ups and downs	1.07	0.40–2.85	.89
An organized religious or spiritual community is important to me	0.82	0.32–2.10	.67
Individual self-care practices (e.g., meditation, yoga, listening to music, exercising, and nature) are important to me	1.28	0.40–4.13	.68
**Depersonalization domain “I have become more uncaring towards people since I took this job”**			
Spiritual well-being is important for one’s emotional well-being	2.01	0.58–6.97	.27
Religious or spiritual beliefs act as a source of comfort and strength during life’s ups and downs	1.84	0.62–5.44	.27
An organized religious or spiritual community is important to me	0.99	0.37 – 2.68	.99
Individual self-care practices (e.g., meditation, yoga, listening to music, exercising, and nature) are important to me	1.13	0.34 – 3.73	.84
**Personal accomplishment domain “If given the opportunity to revisit my career choice, I would choose to become an infection preventionist again”**			
Spiritual well-being is important for one’s emotional well-being	***5.12***	***1.80–14.55***	***.002***
Religious or spiritual beliefs act as a source of comfort and strength during life’s ups and downs	***3.43***	***1.36–8.64***	***.009***
An organized religious or spiritual community is important to me	***4.37***	***1.73–11.02***	***.002***
Individual self-care practices (e.g., meditation, yoga, listening to music, exercising, and nature) are important to me	2.56	0.91–7.24	.08

## Discussion

Our study has several implications. First, it is notable, but unsurprising, that in a heavily Buddhist country such as Thailand (over 90% of the population is estimated to be Buddhist),^9^ self-reported spiritual well-being as well as deeply held religious and spiritual beliefs are integral in the ability of Thai IPs to manage life’s challenges. We might assume that these beliefs therefore may have helped them mitigate the uncertainty of clinical work that was part of everyday life during the height of the COVID-19 pandemic. Second, we identified measures of spirituality and religiosity to have significant associations with IPs’ sense of personal accomplishment (i.e., career satisfaction) during the pandemic. Third, despite no significant associations between measures of religiosity and spirituality and emotional exhaustion (e.g., burnout), a large proportion of Thai IPs indicated that an organized religious or spiritual community was important for their spiritual well-being. Taken together, these findings suggest that mechanisms for supporting spirituality during clinical practice could help improve some domains of well-being among IPs.

Our study has several limitations, including a modest sample size of lead IPs at 100 hospitals, which limited our ability to examine associations between IP measures of spirituality, religiosity, self-care, and well-being. In addition, the 100% response rate may imply that these hospitals systematically differ from the hospitals we did not incorporate in our convenience sample. However, our convenience sample was nationally representative, with a diverse mix of hospital types from all regions in Thailand. Since the primary focus of our survey was to assess what Thai hospitals are currently doing to prevent common healthcare-associated infections,^2^ we did not assess IP well-being using more comprehensive measures of well-being or burnout. However, our survey did include single-item questions representing the primary burnout domains of emotional exhaustion, depersonalization, and personal accomplishment which have demonstrated reasonable reliability.^10^ Despite these limitations, our findings suggest that spirituality, religion, and self-care likely play important roles in promoting well-being among IPs in Thailand.

To our knowledge, this is the first study to explore the association between spirituality, religious beliefs, and well-being among IPs in a largely Buddhist country. We found a state of well-being, spirituality, religiosity, and self-care to be important factors for IPs’ feelings of career satisfaction during the COVID-19 pandemic. Recognizing—and perhaps even facilitating—spiritual, religious, and self-care practices within the clinical setting may enhance emotional well-being among Thai healthcare workers.
